# Effect of the COVID-19 pandemic on cardiac arrest resuscitation practices and outcomes in non-COVID-19 patients

**DOI:** 10.1186/s40560-021-00570-8

**Published:** 2021-09-10

**Authors:** Sau Ki Tong, Lowell Ling, Jack Zhenhe Zhang, Florence H. Y. Yap, Kam Leung Law, Gavin M. Joynt

**Affiliations:** 1 Department of Anaesthesia and Intensive Care, 4/F Main Clinical Block and Trauma Centre, Prince of Wales Hospital, Shatin, Hong Kong SAR, China; 2 Department of Anaesthesia and Intensive Care, The Chinese University of Hong Kong, Hong Kong SAR, China; 3 Accident and Emergency Department, North District Hospital, Hong Kong SAR, China

**Keywords:** Cardiopulmonary resuscitation, 2019 Novel Coronavirus disease, Pandemic, Outcome, Indicator, Return of spontaneous circulation, Team, Resuscitation team, Cardiac arrest

## Abstract

The effect of changes to cardiopulmonary resuscitation (CPR) procedures in response to Coronavirus disease 2019 (COVID-19) on in-hospital cardiac arrest (IHCA) management and outcomes are unreported. In this multicenter retrospective study, we showed that median time to arrival of resuscitation team has increased and proportion of patients receiving first-responder CPR has lowered during this pandemic. IHCA during the pandemic was independently associated with lower return of spontaneous circulation OR 0.63 (95% CI 0.43–0.91), despite adjustment for lowered patient comorbidity and increased time to resuscitation team arrival. Changes to resuscitation practice in this pandemic had effects on IHCA outcomes, even in patients without COVID-19.

Outcomes of in-hospital cardiac arrest (IHCA) in patients with Coronavirus Disease 2019 (COVID-19) are poor [1]. Furthermore, changes to cardiopulmonary resuscitation (CPR) procedures have been recommended to reduce provider exposure to COVID-19 cross-infection [2]. These include donning personal protective equipment (PPE) prior to CPR, limiting personnel numbers, and determining appropriate CPR thresholds. The effects of these changes made in response to the pandemic on cardiac arrest management and outcomes are unreported.

This retrospective analysis of prospectively collected audit data at two public Hong Kong hospitals compared the outcomes of adult IHCA who received CPR before and during the COVID-19 pandemic. Two 1-year periods: January 27, 2019 to January 26, 2020 (pre-COVID-19) and January 27, 2020 to January 26, 2021 (COVID-19) were compared. January 27, 2020 corresponds with the first reported critical case of COVID-19 in the territory. The primary objective was to compare time from arrest recognition to CPR, and arrival of resuscitation team. The secondary objective was to identify patient and management factors associated with return of spontaneous circulation (ROSC). The study was approved by the Joint CUHK-NTEC Clinical Research Ethics Committee (2021.212).

During the study period there were 693 adult IHCA who received CPR, of which 630 were first cardiac arrest episodes. We analyzed the outcomes of 629 IHCA patients after excluding one patient with undocumented arrest time. The IHCA incidence rates were 1.6 and 1.37 per 1000 admissions before and during the pandemic, respectively (*P* = 0.017). Patient characteristics are summarized in Table 1. No study patients had COVID-19. The results of univariate and multivariate analysis for factors associated with ROSC are shown in Table 2.

**Table 1:** Patient characteristics and outcomes

	Pre-COVID-19 *n* = 362	COVID-19 *n* = 267	*P* value
Median age, years (IQR)	76 (66–85)	77 (66–85)	0.689
Age, years			
≤ 65	90 (25)	64 (24)	0.888
66–75	87 (24)	58 (22)	
76–85	99 (27)	81(30)	
≥ 86	86 (24)	64 (24)	
Male sex (%)	240 (66)	154 (58)	0.027
Charlson comorbidity index			
0 (%)	110 (30)	103 (39)	0.024
1–2 (%)	126 (35)	96 (36)	
≥ 3 (%)	126 (35)	68 (26)	
Median interval between admission to arrest, day (IQR)	3 (1–10)	2 (1–8)	0.056
First-responder CPR (%)	290 (80)	194 (73)	0.028
Immediate resuscitation team arrival	116 (32)	61 (23)	0.014
Median resuscitation team arrival time^a^, min (IQR)	2 (0–5)	3 (1–6)	0.005
Witnessed arrest^a^ (%)	239 (66)	167 (63)	0.401
Monitored area (%)	46 (13)	39 (15)	0.491
Shockable rhythm* (%)	30 (9)	19 (7)	0.358
ROSC^a^ (%)	133 (37)	73 (28)	0.014
Duration of CPR, min (IQR)	22 (12–33)	21 (12–34)	0.697
Hospital survival (%)	16 (4)	14 (5)	0.632

Team arrival time was defined as the first documented arrival time of a designated resuscitation team member at each hospital. First-responder CPR was defined as immediate commencement of CPR within 1 min of documented cardiac arrest. Immediate resuscitation team arrival was defined as arrival of the resuscitation team within 1 min of documented cardiac arrest. Median time to commencement of CPR was not calculated because > 50% of the documented times to CPR was less than 1 min. ^a^Missing data: 8 for arrival time of resuscitation team, 1 for witnessed arrest, 15 for shockable rhythm, 3 for ROSC. *CPR* cardiopulmonary resuscitation, *COVID-19* coronavirus disease 2019, *IQR* interquartile range, *ROSC* return of spontaneous circulation

**Table 2:** Factors associated with ROSC

	Univariate		Multivariate	
	Odds ratio (95% CI)	*P* value	Odds ratio (95% CI)	*P* value
Age	0.98 (0.97–0.99)	< 0.005	0.99 (0.97–1.00)	0.025
Charlson comorbidity index	1.00 (0.93–1.08)	0.996	–	
Male gender	1.12 (0.79–1.59)	0.518	–	
COVID-19 pandemic	0.65 (0.46–0.92)	0.015	0.63 (0.43–0.91)	0.015
Witnessed arrest	2.71 (1.85–4.00)	< 0.005	2.06 (1.34–3.16)	0.001
Monitored area	3.06 (1.92–4.89)	< 0.005	2.07 (1.24–3.44)	0.005
Shockable rhythm	3.00 (1.66–5.43)	< 0.005	2.01 (1.06–3.82)	0.034
First-responder CPR	1.20 (0.80–1.80)	0.376	–	
Immediate resuscitation team arrival	2.19 (1.53–3.15)	< 0.005	1.53 (1.03–2.29)	0.035

Logistic regression was used to perform univariate analysis. Generalized linear mixed model with multinomial logistic regression and hospital as random effects was used for multivariate analysis. These analyses were performed on 626 patients because 3 patients in the cohort had missing ROSC data. *CPR* cardiopulmonary resuscitation, *COVID-19* coronavirus disease 2019, *ROSC* return of spontaneous circulation

Our results are consistent with a single-center study which showed lower IHCA survival during the pandemic [3]. However, inclusion of COVID-19 patients may have biased their conclusions since COVID-19 patients with IHCA rarely survive to discharge [1, 4]. In contrast, our study is multicenter, of larger size, and compared two 1-year periods, before and during COVID-19. During this pandemic, Hong Kong’s healthcare system has been challenged but not overwhelmed as occurred in other regions. Mortality of COVID-19 remains low at 1.8% [5]. Importantly, lack of COVID-19 patients in this study removes its potential as a confounder for IHCA outcomes. Thus, we could assess the effect of resuscitation practice changes on patient characteristics, timing of IHCA management and association with ROSC.

The incidence of IHCA and comorbidity among those who received CPR were lower during the pandemic, possibly due to proactive do-not-resuscitate order discussions [2]. Since the pandemic, our IHCA resuscitation teams were instructed to don respiratory PPE prior to CPR regardless of patients’ COVID-19 status or before arrival to isolation wards. The rationale was that patients may have asymptomatic disease and some patients with initial negative tests at admission may turn positive during their hospital stay. Therefore, the approach of first responders and resuscitation teams to PPE preparation was consistent even though they may not have known whether the arrested patient had COVID-19 at the time of arrest. Our data show this has increased resuscitation team arrival time and lowered the proportion of patients receiving first-responder CPR. Furthermore, we showed that arrest during the pandemic was independently associated with lower ROSC. This was despite adjustment for lowered patient comorbidity, increased time to resuscitation team arrival, and other well-established factors associated with ROSC, such as shockable rhythm.

We used ROSC rather than hospital mortality as our endpoint because of the low 5% survival. Although a limiting factor, survival rates were consistent with reported regional data [6]. We were also unable to assess changes in airway management during CPR as the data were inconsistently recorded. Nevertheless, our study demonstrated changes to resuscitation team practice in response to the pandemic had effects on IHCA outcomes, even in patients without COVID-19.

## Data Availability

The datasets used and/or analyzed during the current study are available from the corresponding author on reasonable request. Approval from The Joint Chinese University of Hong Kong—New Territories East Cluster Clinical Research Ethics Committee will be required before sharing of data.

## Abbreviations

COVID-19: Coronavirus disease 2019
IHCA: In-hospital cardiac arrest
ROSC: Return of spontaneous circulation
CPR: Cardiopulmonary resuscitation
PPE: Personal protection equipment
